# Secondary Syphilis of Middle Ear; Unilateral Facial Paralysis

**Published:** 1887-07

**Authors:** S. Latimer Phillips

**Affiliations:** Savannah, Ga., Formerly House Surgeon to the Presbyterian Eye, Ear, and Throat Charity Hospital, Baltimore, Md.


### SECONDARY SYPHILIS OF MIDDLE EAR; UNILATERAL FACIAL PARALYSIS.

By S. LATIMER PHILLIPS, M. D., SAVANNAH, GA.,

Formerly House Surgeon to the Presbyterian Eye, Ear, and Throat Charity Hospital, Baltimore, Md.

"In some instances syphilis apparently causes well marked changes in the middle ear, which alterations have been very erroneously referred to the nerve-structures of the internal ear, especially to the cochlea. There are many more reasons for placing these apparently syphilitic changes in the tissues of the middle ear, the conductive functions of which we are acquainted with, than in the labyrinthine and nervous structures of the mechanism of which physiologists know nothing positive." So says Dr. C. H. Burnett.* I cannot think but that this is true ; that many cases

* A Treatise on the Ear, second edition, 1884, p. 389.

of sudden deafness in syphilitic patients have been ascribed to lesions of the internal ear, when, if a more thorough and systematic examination had been made, the true cause would have been found in some change in the middle ear or tympanic cavity. He goes on further to say : "These cases of syphilitic deafness, due to change in the middle ear, are characterized by the suddenness and profundity of the hardness of hearing." In support of this I repoit the following case :

On November 8, 1886, H. L. G——, aged twenty-five, was sent to me by Dr. George H. Stone, of this city, for treatment of double iritis and hardness of hearing. I got the following history from him : Three months since had sore on glans penis, with chain of enlarged glands in groins. He was treated with mercury by Key's tonic method, and with iodide of potash. He did well, and soon chancre had healed. He now ceased to take his medicine, notwithstanding he had been advised to the contrary. Six or seven weeks after the healing of the sore, he says he had general paralysis, from which he soon recovered. One week after onset of paralysis suffered severely from pain in head, which was augmented as night came on. At this time he became suddenly deaf, but at no time has he had any pain in his ear. Shortly after his deafness found that, in eating, the food would collect between the teeth and cheeks on the left side ; could not purse up lips to whistle, and that face on that side had lost its expression. I still find this the case, but he tells me it is not as bad as it was (November 8); could not shut eyelids of left side tightly together ; tongue, when protruded, did not deviate to either side ; no paralysis of uvula ; and stated that he had no loss of taste on the affected side. Hearing on the right side reduced to ticking of watch at 2½ inches ; left side watch tick at 1½ inches ; external auditory canals normal ; no redness about them, as mentioned by Sexton and Bumstead in syphilitic ear troubles. On inspecting membranes they were found to be somewhat sunken, particularly the left, with very small light spots ; left manubrium congested along its course. Both membranii tympani had "ground glass" appearance or tint ; cavities easily and freely inflated with eustachian catheter, as well as with Poletzer's method. Tuning-fork, when placed on vertex, was heard equally well on either side, and, when the meatus was closed with the finger, was heard better on that side. No ulceration about throat ; no granulomata of pharynx ; but a little catarrhal inflammation of little import, as he did not complain at this time of any throat symptoms ; cervical glands not enlarged. He was put on large and increasing doses of iodide of potash and mercury by inunction, it having disagreed with him when administered through the stomach. Under this treatment, with daily inflation of tympanic cavities, through the eustachian catheter, he began to improve, and continued to do so until he passed from under my observation, in December.

At that time, hearing in right ear had been brought up to 10 inches; in left, 5½ inches. Paralysis had almost entirely disappeared, a slight shade only remaining, indicated by a little drawing of the muscles to the right side.

The two most important points in this case are: The situation of the trouble, and its nature. With the tuning-fork—by tone conduction—we eliminate all idea of the lesion being situated in the nerve apparatus of labarynth; with no throat disease to speak of, a thoroughly patulous condition of the tubes, and the appearance of the drum membranes, we are compelled by force of circumstances to place the seat of the changes in the tympanic cavities. As to its nature, Dr. Sexton tells us: "That it may be surmised that granulomo, or circumscribed, small, round cell-infiltration takes place within the tympanum; that the invasion is rapid, and that it prevents, by fixation, the conductive apparatus from its normal movements." * Under the influence of mercury and iodide of potash, with the local treatment of inflexion, which has a tendency to break up any adhesions which may have formed in the tympanæ, it may be supposed that these "granuloma" gradually underwent diminution in size, consequently a freer movement of the ossicula, and improved hearing power.

